# Mental health from childhood to adolescence predicts excessive weight and body composition at 18 years

**DOI:** 10.1016/j.nut.2024.112527

**Published:** 2024-10

**Authors:** Iná S. Santos, Isabel O. Bierhals, Luciana Tovo-Rodrigues, Aluísio JD Barros, Tiago Munhoz, Marina Xavier Carpena, Alicia Matijasevich

**Affiliations:** a Post-graduate Program in Epidemiology, Federal University of Pelotas, Pelotas. Rio Grande do Sul, Brazil; b School of Psychology, Federal University of Pelotas, Pelotas. Rio Grande do Sul, Brazil; c Departamento de Medicina Preventiva, Faculdade de Medicina FMUSP, Universidade de São Paulo, São Paulo, Brazil

**Keywords:** Mental health, Body composition, Body mass index, Ultra-processed foods, Cohort studies

## Abstract

**Objective:**

To investigate the association of mental health in childhood and adolescence with four outcomes at 18 years: ultra-processed food (UPF) consumption, body mass index (BMI), excessive weight (EW), and body composition, including fat mass (FM) and fat free mass (FFM) in kg, FM index (FMI) and FFM index (FFMI) in kg/m^2^.

**Methods:**

Cohort study in which The Development and Well-Being Assessment (DAWBA) (6 and 11 years) and the MINI International Neuropsychiatric Interview (MINI) (18 years) provided information on internalizing (INT), externalizing (EXT) and any mental disorder (ANY). The exposure was classified in: “never”, “at 6 and/or 11 years”, “at 18 years only” and “at 6, 11, and 18 years”. Linear and logistic regression were run. All analyses were stratified by sex.

**Results:**

A total of 2722 participants were analyzed. At 18 years, female with EXT disorders at 6 and/or 11 years presented higher BMI (β: 1.70; 0.18–3.23), FM (β: 4.74; 1.42–8.06), and FMI (β: 1.53; 0.28–2.79) than those who never had. The odds of EW at 18 years was also higher in females with EXT disorders at 6 and/or 11 years (OR: 3.39; 1.56–7.36) and at the three time points (OR: 7.08; 1.69–29.59). Males with EXT disorders at 6 and/or 11 years presented higher FM (β: 4.45; 1.85–7.06) and FMI (β: 1.47; 0.63–2.31).

**Conclusions:**

Among children and adolescents showing symptoms of EXT disorders, weight should be monitored carefully, thus ultimately contributing to reduce the burden of EW in adolescence.

## Highlights

• Female with EXT disorders at 6 and/or 11y presented higher BMI, FM, and FMI at 18 years.
• The odds of EW was higher in females with EXT disorders at 6 and/or 11 years.
• The odds of EW was also higher in females with EXT disorders at 6, 11 and 18 years.
• Males with EXT disorders at 6 and/or 11 years presented higher FM and FMI.

## Introduction

Overweight and obesity in childhood and adolescence are associated with adverse health consequences throughout the life-course. Gaining excess weight in childhood and adolescence is likely to lead to lifelong overweight and obesity [1]; and being overweight in childhood and adolescence is associated with greater risk and earlier onset of chronic disorders such as type 2 diabetes [2].

Although obesity may be driven by several factors like heritability, early-life undernutrition, gut microbiome, environmental contaminants, sleep deprivation, and chronic stress [3], conventionally it is thought to be a consequence of prolonged caloric intake in excess of energy requirements. In this way, obesity may partly result from psychological factors affecting energy intake and expenditure by altering hormones and neurotransmitters that influence weight gain and food intake [4], triggering eating to regulate affect [5], and decreasing physical activity [6]. The regulation of food intake and eating behavior is complex and involves three areas of the brain interacting with each other [7]. The first is the hypothalamus, in response to hormonal signals from the digestive tract and adipose tissue. The second is the rewards system, in which the processes of motivation to seek reward, learning, and the consolidation of eating behavior arise (external stimuli such as emotions play a significant role in triggering these behaviors). The third is the prefrontal cortex functions, including self-regulation control of eating behaviors. Greater negative affect and lower positive affect are associated with greater emotional eating (stress eating), defined as the propensity to eat in response to positive and negative emotions and not physical need [8]. Nonetheless, the causal nature of the relationship between excessive weight and mental health remains uncertain [9,10].

A literature review with the aim of assessing the association between diet quality and mental health in childhood or adolescence concluded that this area had a limited body of evidence, largely attributable to a dearth of prospective and case-control data, which thereby precludes inferring causal associations about these relationships [11]. Prospective studies offer an important opportunity for an in-depth analysis of the relationship between mental health and obesity. Among observational epidemiological designs, prospective birth cohorts are the best approach to analyzing such associations. Therefore, given the public health importance of increasing rates of both obesity and common mental disorders [12,13] and the scarcity of longitudinal studies on populations from middle-income countries such as Brazil (which, together with 9 other countries shelters half of the world's obese people) [3], the aim of this study was to investigate the association of mental health (as the exposure) in childhood and adolescence with four outcomes at 18 years of age: ultra-processed food (UPF) consumption, body mass index (BMI), excessive weight, and body composition.

## Material and methods

### Sample

The Pelotas 2004 Birth Cohort is a longitudinal population-based study, initiated with newborns from hospital births to mothers residing in the urban area of Pelotas, a municipality with around 325,000 inhabitants in the south of Brazil [14]. From January 1st to December 31st of 2004, all hospitals with maternity wards were visited daily, and all live births (N = 4263) were eligible for enrollment in the cohort [15,16]. The mothers of 4,231 newborns agreed to take part in the study. Newborns were examined by the research team in the hospital at the first 24 hours postpartum (perinatal study). To date, cohort members have been followed-up on several occasions at mean (standard deviation) ages of 3.0 (0.1), 11.9 (0.2), 23.9 (0.4), and 49.5 (1.7) months, and at 6.8 (0.3), 11.0 (0.3), 15.7 (0.2), and 18.0 (0.3) years. The retention rate was greater than 90% at each follow-up up to age 11; approximately 50% at age 15 due to the interruption of data collection at the research clinic because of the COVID-19 pandemic; and greater than 80% at age 18.

The current study included participants with available information on the exposure of interest (mental disorders) and outcomes (UPF consumption, BMI, excessive weight, and body composition parameters) in the follow-up at 18 years old in 2022. Also, data from the perinatal study and from the 3-month and the 4-, 6-, and 11- years follow-ups (conducted respectively in 2004, 2008, 2010, and 2015) were used. Up to the 48-mo follow-ups, the participants were assessed in their homes. From the 6-year follow-up onwards, the assessments were carried out in a clinic specifically structured for the research.

## Outcomes

All outcomes were assessed at the 18-year follow-up.

### UPF consumption

The UPF consumption (in grams) assessment was performed using a computer-based semi-quantitative food frequency questionnaire (FFQ) consisting of 94 food items. The instrument was self-applied by the participant and the recall period was the previous 12 months. For each food item, the number of times it was consumed (for a day, week, month, or year) and the size of the portion consumed (lower, equal to or higher than the average portion) was questioned. Based on domestic measures, the average portion sizes were defined according to the Table for Assessment of Food Intake in Household Measures [17], presented to the respondents with the help of images.

The frequency of consumption of each food item was first converted into annual consumption. For this, the frequencies were multiplied by zero, 12, 52, 104, 260, 365.25, 730.5, and 1826.25, according to the answer options: never or <1 time a month, 1–3 times a month, once a week, 2–4 times a week, 5–6 times a week, once a day, 2–4 times a day, and ≥ 5 times a day, respectively [18]. With the daily frequency consumption and the portion size, the quantity in grams of each food was calculated. The FFQ items were classified in the four groups proposed by the NOVA classification (unprocessed or minimally processed foods, processed culinary ingredients, processed foods, and ultra-processed foods - UPF) [19]. A total of 26 food items were classified as UPF. For statistical analysis, daily UPF consumption in grams was categorized into tertiles according to the sample distribution, and subsequently the variable was dichotomized in highest tertile of daily UPF consumption (yes or no).

### BMI and excessive weight

For the BMI (kg/m^2^) calculation, the body weight was measured with a high-precision scale with 0.01 kg resolution (model BWB-627-A, Tanita, Tokyo, Japan) coupled to a BODPOD machine. The height was collected by trained anthropometrists, applying standardized technique, with the help of a Harpenden portable stadiometer with maximum height of 2.06 meters and 1 mm of accuracy. The BMI was calculated by dividing the weight in kg by the height in squared meters. Excessive weight (overweight and obesity) was defined as BMI ≥ 25 kg/m^2^ [20].

### Body composition

Fat mass (FM) and fat-free mass (FFM) in kg were obtained by air-displacement plethysmography (BODPOD), that provides a direct estimation of this measures. Standard equation was used to define FM and FFM, according to age and sex [21]. Values in kg were also converted into an index relative to the height. The FM index (FMI) and FFM index (FFMI) parameters were calculated by dividing the values in kg by the height in squared meters.

### Exposure of interest

The exposure of interest was any mental disorder (ANY), internalizing (INT) and externalizing (EXT) disorders. At the 6- and 11-year follow-ups, mental health was measured through the Development and Well-Being Assessment (DAWBA) [22], via interviews with primary caregivers (97% mothers), and at the 18-year follow-up it was assessed using the Mini International Neuropsychiatric Interview (MINI), version 7.0.2, directly applied to the adolescent [23]. The DAWBA and MINI are structured diagnostic interview based on DSM-IV, DSM-5 and ICD-10 diagnoses [24], that have previously been translated and validated for use in Brazil [25,26]. Interviews were conducted by trained psychologists with approximately 40 hours of training delivered by an experienced child psychologist with a background in epidemiological assessment. Additionally, weekly supervision was provided throughout data collection.

The INT disorder includes the occurrence of any anxiety (separation anxiety disorder, specific phobia, social phobia, panic disorder, agoraphobia, posttraumatic stress disorder, obsessive-compulsive disorder, generalized anxiety disorder, and another anxiety disorder) or any mood (disruptive mood dysregulation disorder - DMDD, major depression, other depression, mania/bipolar). The EXT disorder includes the occurrence of any attention deficit hyperactivity disorder - ADHD (ADHD combined, ADHD inattentive, ADHD hyperactive-impulsive, and other hyperactivity) or any disruptive disorder (oppositional defiant disorder - ODD, conduct disorder - CD, and other disruptive disorder). “Any disorder” includes INT and EXT disorders in addition to other disorders not classified as INT or EXT, that were present in both instruments.

A chronicity variable of having ANY, INT and EXT disorders was created, based on the presence of symptoms in the 6-, 11- and 18-year follow-ups. Because symptoms were more prevalent at 18 than in the earlier ages, the variable was arranged in four categories as: “never,” “at 6 and/or 11 years,” “only at 18 years,” and “at 6, 11, and 18 years.”

### Potential confounders

Potential confounders comprised maternal and child characteristics at birth, 3 months, 4 and at 6 years of life. From the mother the following characteristics were employed: age (≤24, 25–34, ≥35 years), education (0–4, 5–8, ≥9 completed years of formal education), parity (<2, ≥2), and total monthly family income in quintiles (the first quintile comprised the poorest families and the fifth quintile the wealthiest).

Characteristic of the child at birth included intrauterine growth (birth weight according to gestational age and sex) assessed using the INTERGROWTH-21 parameters, and considering newborns small for gestational age (SGA; birth weight below the 10th percentile), appropriate for gestational age (AGA; birth weight between the percentile 10 and 90), and large for gestational age (LGA; birth weight above the 90th percentile) [27]. Breastfeeding pattern (categorized into non-breastfeeding, partial breastfeeding, predominant breastfeeding, and exclusive breastfeeding) was obtained at the 3-month follow-up, and the duration of any breastfeeding (as a continuous variable in months) was collected at the 4-year follow-up.

From the 6-year follow-up the following characteristics were used: the adolescent global intelligence quotient (IQ), ethnicity, screen time (≤3, >3 h/d watching TV), and physical activity. The IQ was assessed by means of the Wechsler Intelligence Scale for Children-III (WISC-III) validated for the Brazilian population [28], and applied by trained psychologists at the 6-year follow-up. The test was composed of four subtests: two verbal (similarities and arithmetic) and two performances (block building and picture completion). A short-form version of the scale was used because of time constraints as a large number of children had to be evaluated. This version was developed by Kaufman [29] and showed a correlation above 0.90 with IQ measured by the full scale. Score conversion tables for the U.S. population were used to calculate IQ scores from the subtests.

As based on maternal response, the participant ethnicity was categorized as black, brown, white, yellow, or indigenous, according to the classification adopted by the Brazilian Census Bureau [30]. Because children in the black, brown, yellow, or indigenous categories had similar socio-demographic characteristics they were assembled into a single group and the variable was categorized into two groups: “White” and “Black/brown, yellow, indigenous”.

Physical activity was determined by accelerometers (GENEActiv; ActivInsights, Kimbolton, UK and Actigraph GT3X), used by the participants for 4–7 days with a 24-hours protocol. The raw data were analyzed with R-package GGIR 2.2-0 [31]. The variable used was the daily average time spent in moderate to vigorous activities for at least 10 minutes, expressed in minutes (cut-off point of 100 mG, an acceleration threshold corresponding to the walk; and 10-minutes bout). Quartile cut-off was set to classify participants as very low active, low active, active, and very active, according to the levels defined by the Institute of Medicine (IOM) [32].

### Data analyses

Data were collected and entered directly in software Pendragon and REDCap (Research electronic data capture) [33]. The statistical package Stata version 16.0 (College Station, TX: StataCorp LLC. StataCorp. 2017) was used to run the analyses. The descriptive analysis was based on calculation of the absolute and relative frequencies of the variables. The chi-square heterogeneity test was used to compare characteristics at birth of the participants included in the analyses with the original sample. Mean BMI, body composition parameters and daily UPF consumption (g), as well the prevalence of excessive weight, any disorder, INT, and EXT disorders at ages 6, 11, and 18 years, with 95% confidence intervals (95%CI) were calculated. The prevalence of each category of the chronicity of any disorder, INT and EXT disorders was also described.

To assess the association between variation in the chronicity of having ANY, INT and EXT disorders and the outcomes BMI, FM, FFM, FMI, and FFMI unadjusted and multiple linear regression were run and beta (β) coefficient with 95%CI were calculated. For the outcomes highest tertile of daily UPF consumption and excessive weight, crude and adjusted logistic regression were performed and odds ratios (OR) with 95%CI were obtained. P-values for heterogeneity were calculated. The “never” group was adopted as the category of reference for unadjusted and adjusted analyses. All covariables were entered in the multivariable model according to a hierarchical model defined by the authors, irrespective of the level of statistical significance of the association with the outcome on bivariate analysis. As recommended for the retention of confounding variables in a model, after allowing for variables at the same level and from higher levels, variables associated with the outcome at p-value ≤ 0.20 were kept in the model [34].

The first level comprised family income, maternal schooling, maternal age, and parity; the second level was composed of ethnicity and intrauterine growth; level 3 consisted of breastfeeding pattern at three months; level 4 of breastfeeding duration; and level 5 of physical activity, screen time and IQ at 6 years. For each outcome, the adjusted model included the measure of the outcome variable collected at the 6-year follow-up (period of onset of the exposure of interest). For instance, when the outcome was the highest tertile of daily UPF consumption, the multivariable model included the mean daily UPF consumption at 6 years of age, when the outcome was excessive weight, excessive weight at 6 years (yes or not) was included, and son on for the remaining outcomes. The UPF consumption at 6 years was assessed using a computer-based semi-quantitative FFQ consisting of 54 food items, answered by the mothers, with a recall period for the last 12 months. Classification of the foods according to the level of food processing, and calculation of daily consumption of UPF followed the same methodology used at the 18-year follow-up described above. At 6 years, BMI z-scores specific for sex and age were calculated according to the growth curves published by the World Health Organization (WHO) in 2007 [20], using ANTHRO PLUS software downloaded from the WHO website [35]. Excessive weight (overweight and obesity) was defined as BMI-for-age ≥ +1 z-score [20]. Body composition parameters (FM, FFM, FMI, and FFMI) at 6 years were estimated by air-displacement plethysmography with the same equipment used at 18 years but employing equations suitable for the age of 6 years [36].

Considering the difference in the occurrence of exposures and outcomes between males and females, all analyzes were stratified by sex. All assumptions for generalized linear models and for goodness-of- fit of logistic regression models were tested and the modeling used showed to be sensitive to the research question. The significance level adopted for the two-tailed testing was *p* < 0.05.

To check for reverse causality, that is, whether the adolescents had experienced mental health disorders because they were living with obesity, ate too many processed foods and so on, we repeated the analyses taking ANY, INT and EXT disorders as outcomes and UPF consumption, BMI, excessive weight FM, FFM, FMI, and FFMI as the exposures. For these analyses, UPF consumption, BMI, FM, FFM, FMI, and FFMI at 6, 11, and 18 years were dichotomized using the highest tertile of the sample distribution as the cut-off point (highest tertile; yes or no). Excessive weight at 6, 11, and 18 years was defined as yes or no. Crude and adjusted logistic regression was used to calculate odds ratios with 95%CI. Also, to explore the temporal sequence between mental disorders and the outcomes in normal weight children, supplemental analyses were run after excluding those with excessive weight at 6 or 11 years.

### Ethics approval

The study protocol and all follow-ups of the Pelotas 2004 Birth Cohort were approved by the Research Ethics Committee of the Faculty of Medicine of the Federal University of Pelotas. The approval protocol number of the 18-year follow-up was 5.210.484 and that of the Presentation Certificate for Ethical Appreciation (CAAE) was 54362821.0.0000.5317. In all follow-ups informed consent was obtained in writing from the mothers or legal guardians. At 11 and 18 years old, the participants also signed a free and informed consent form.

## Results

A total of 2722 cohort participants were included in the analyses. The comparison between the analyzed sample and the whole cohort at the perinatal study is shown in Table 1. Except for maternal education (the proportion of mothers with 0-4 years of formal education was lower in the analyzed sample than in the whole cohort; *p* = 0.043), the distribution of all other maternal variables and the participants perinatal characteristics were similar in the analyzed sample and in the entire cohort.

**Table 1:** Description of the original sample and participants included in the analysis. The 2004 Pelotas Birth Cohort, Brazil

Variables	Original sample (N = 4231)	Included sample (N = 2722)	*P-value*^a^
	N (%)	N (%)	
*Maternal perinatal characteristics*			
Family income at birth (quintiles)			0.061
1° (lowest)	871 (20.6)	489 (18.0)	
2°	854 (20.2)	547 (20.1)	
3°	816 (19.3)	546 (20.1)	
4°	858 (20.3)	602 (22.1)	
5° (highest)	830 (19.6)	538 (19.7)	
Maternal age at birth (years)			0.151
≤ 24	1947 (46.1)	1194 (43.9)	
25-34	1717 (40.6)	1133 (41.6)	
≥ 35	563 (13.3)	394 (14.5)	
Maternal education at birth (years)			0.043
0-4	654 (15.6)	368 (13.7)	
5-8	1731 (41.3)	1104 (41.0)	
≥ 9	1801 (43.0)	1221 (45.3)	
Parity			0.131
< 2	1665 (39.4)	1095 (40.2)	
≥ 2	2563 (60.6)	1627 (59.8)	
Depression or nerve problems during pregnancy			0.153
No	3168 (75.0)	2080 (76.5)	
Yes	1059 (25.0)	640 (23.5)	
*Participants perinatal characteristics*			
Sex			0.314
Male	2195 (51.9)	1378 (50.6)	
Female	2036 (48.1)	1344 (49.4)	
Ethnicity			0.576
White	2726 (68.2)	1837 (67.5)	
Black, brown, yellow, indigenous	1272 (31.8)	884 (32.5)	
Intrauterine growth			0.930
Small for gestational age	350 (8.6)	220 (8.3)	
Apropriate for gestational age	3023 (74.2)	1965 (74.5)	
Large for gestational age	702 (17.2)	452 (17.2)	

BMI: body mass index; N: number of participants; %: percentage.

a *p*-value refers to Chi-squared heterogeneity test.

Table 2 presents the mean BMI and body composition parameters, and the prevalence of excessive weight and of the highest tertile of daily UPF consumption at 6, 11, and 18 years, stratified by sex. At 6 and 18 years, FM and FMI means were higher in females than in males, whereas FFM and FFMI were higher in males. There was no difference between sexes for mean BMI and prevalence of excessive weight at 6 years. On the other hand, at 18 years mean BMI and prevalence of excessive weight were higher in female than in male adolescents. At 11 years there was a different pattern, with females presenting higher FFM and lower prevalence of excessive weight than males, and no difference between sexes in terms of FFMI or FM. The prevalence of being in the highest daily UPF consumption was higher in females than in males at the three time points.

**Table 2:** Body composition parameters and daily ultra-processed food of the analyzed sample at 6, 11 and 18 years of age. (N = 2722)

Variable	Males (N = 1378)		Females (N = 1344)	
	Mean	95%CI	Mean	95%CI
*At 6 years*				
Fat mass (kg)	6.0	5.8-6.2	6.7	6.5-6.9
Fat-free mass (kg)	19.3	19.1-19.4	18.3	18.1-18.4
Fat mass index (kg/m^2^)	4.0	3.8-4.1	4.5	4.4-4.7
Fat-free mass index (kg/m^2^)	13.0	12.9-13.0	12.5	12.5-12.6
Body mass index (kg/m^2^)	16.9	16.8-17.1	17.1	16.9-17.2
	%	95%CI	%	95%CI
Excessive weight^1^	35.5	32.9-38.2	37.3	34.7-40.0
Highest tertile of daily UPF consumption	28.8	26.4-31.4	37.0	34.4-39.7

	Mean	95%CI	Mean	95%CI
*At 11 years*				
Fat mass (kg)	11.9	11.5-12.3	12.1	11.7-12.5
Fat-free mass (kg)	30.2	29.9-30.4	31.0	30.7-31.4
Fat mass index (kg/m^2^)	5.5	5.3-5.7	5.5	5.3-5.7
Fat-free mass index (kg/m^2^)	14.3	14.2-14.4	14.3	14.3-14.4
Body mass index (kg/m^2^)	19.8	19.6-20.0	19.9	19.6-20.1
	%	95%CI	%	95%CI
Excessive weight^1^	47.9	45.2-50.5	42.1	39.5-44.8
Highest tertile of daily UPF consumption	31.8	29.4-34.4	37.4	34.8-40.0

	Mean	95%CI	Mean	95%CI
*At 18 years*				
Fat mass (kg)	15.2	14.6-15.8	22.6	22.0-23.3
Fat-free mass (kg)	56.2	55.8-56.7	40.8	40.5-41.1
Fat mass index (kg/m^2^)	5.0	4.8-5.2	8.8	8.5-9.1
Fat-free mass index (kg/m^2^)	18.6	18.5-18.7	15.9	15.7-16.0
Body mass index (kg/m^2^)	23.6	23.3-23.9	24.6	24.3-24.9
	%	95%CI	%	95%CI
Excessive weight^2^	28.4	26.1-30.8	39.1	36.6-41.8
Highest tertile of daily UPF consumption	30.2	27.8-32.7	37.4	34.9-40.0

95%CI: 95% confidence interval; kg: kilograms; kg/m^2^: kilograms per meter squared; UPF: ultra-processed food.

1 BMI≥ +1 z-score for age and sex [20].

2 BMI≥ 25 kg/m^2^.

Table 3 displays the prevalence of ANY, INT, and EXT disorders at 6, 11, and 18 years of age. More than one quarter of the males and about 50% of the females presented any disorder at age 18 years. At 6 and 18 years, in both sexes, the prevalence of INT disorders was higher than that of EXT disorders. There was no difference in prevalence of INT and EXT disorders at 11 years in both sexes.

**Table 3:** Prevalence of any disorder, internalizing and externalizing disorders at ages 6, 11 and 18 years in the 2004 Pelotas Birth Cohort. (N = 2722)

Variables	Age					
	6 years		11 years		18 years	
	Males	Females	Males	Females	Males	Females
	% (95%CI)	% (95%CI)	% (95%CI)	% (95%CI)	% (95%CI)	% (95%CI)
Any disorder	16.6 (14.7-18.6)	13.5 (11.7-15.4)	14.1 (12.3-16.0)	9.9 (8.4-11.6)	28.3 (26.0-30.7)	47.3 (44.7-50.0)
Internalizing disorder	9.2 (7.8-10.9)	9.0 (7.6-10.7)	6.6 (5.4-8.0)	5.6 (4.5-6.9)	18.8 (16.8-21.0)	39.7 (37.1-42.3)
Externalizing disorder	5.1 (4.0-6.4)	2.5 (1.8-3.5)	6.2 (5.1-7.7)	3.4 (2.6-4.5)	14.2 (12.5-16.2)	15.9 (14.0-17.9)

%: percentage; 95%CI: 95% confidence interval.

At 6 and 11 years, ANY disorders were more prevalent among males, but at 18 years prevalence in females was 67% higher than in males (47.3% *vs.* 28.3%). Prevalence of INT disorders that was similar in both sexes at 6 and 11 years, turned two times higher in females than in males at 18 years (39.7% *vs.* 18.8%). On the other hand, the prevalence of EXT disorders, which was two times higher in males than in females at 6 and 11 years, presented similar prevalence at the two sexes at 18 years, although at a higher rate than at the earlier ages.

In Table 4, the proportion of males who have had ANY disorder, INT, and EXT disorders “at 6 and/or 11 years” was higher than the proportion of females. On the other hand, the prevalence of INT disorders was twice as high in females than in males “at 18 years only” (33.0% vs. 15.5%) and “at 6,11 and 18 years” (6.7% vs. 3.3%). In those with persistence of symptoms (“at 6, 11 and 18 years”) the prevalence of EXT disorders was twice as high in males than in females (2.5% vs. 1.2%, respectively).

**Table 4:** Prevalence of any-, internalizing-, and externalizing-disorder, considering the ages of 6, 11 and 18 years, according to sex. (N = 2722)

Disorders	Males		Females	
	N	% (95%CI)	N	% (95%CI)
Any disorder				
Never	761	55.2 (52.6;57.8)	590	43.9 (41.3;46.6)
At 6 and/or 11y	227	16.5 (14.6;18.5)	118	8.8 (7.4;10.4)
At 18y only	261	18.9 (17.0;21.1)	484	36.0 (33.5;38.6)
At 6, 11 and 18y	129	9.4 (7.9;11.0)	152	11.3 (9.7;13.1)
Internalizing disorder				
Never	969	70.3 (67.9;72.7)	725	53.9 (51.3;56.6)
At 6 and/or 11y	150	10.9 (9.4;12.6)	86	6.4 (5.2;7.8)
At 18y only	213	15.5 (13.6;17.5)	443	33.0 (30.5;35.5)
At 6, 11 and 18y	46	3.3 (2.5;4.4)	90	6.7 (5.5;8.2)
Externalizing disorder				
Never	1083	78.6 (76.3;80.7)	1076	80.1 (77.8;82.1)
At 6 and/or 11y	99	7.2 (5.9;8.7)	55	4.1 (3.2;5.3)
At 18y only	162	11.8 (10.2;13.6)	197	14.7 (12.9;16.7)
At 6, 11 and 18y	34	2.5 (1.8;3.4)	16	1.2 (0.7;1.9)

%: percentage; 95%CI: 95% confidence interval.

The unadjusted association between mental health and the outcomes is shown in Table 5. Females who have had ANY disorder at any time, presented increased odds of higher daily consumption of UPF than their counterparts who have never had ANY disorder. There was no association between INT disorders and highest UPF consumption in both sexes.

**Table 5:** Unadjusted analysis of the association between daily UPF consumption (highest tertile vs. intermediate and lowest tertiles), BMI, excessive weight, and body composition at 18 years with any, internalizing and externalizing disorders. (N = 2722)

Disorders		UPF consumption and body composition parameters						
		Highest tertile of daily UPF consumption^a^	BMI (kg/m^2^)	Excessive weight	FM (kg)	FFM (kg)	FMI (kg/m^2^)	FFMI (kg/m^2^)
		OR (95%CI)	β (95%CI)	OR (95%CI)	β (95%CI)	β (95%CI)	β (95%CI)	β (95%CI)
Any disorder	*Males*	*p* = 0.060	*p* = 0.359	*p* = 0.585	*P* =0.483	*P* =0.945	*P* =0.414	*P* =0.611
	Never	Ref.	Ref.	Ref.	Ref.	Ref.	Ref.	Ref.
	At 6 and/or 11y	1.24, (0.90;1.73)	0.70, (-0.07;1.48)	1.23, (0.89;1.71)	1.41, (-0.37;3.19)	-0.08, (-1.22;1.06)	0.50, (-0.08;1.08)	0.20, (-0.11;0.51)
	At 18y only	1.26, (0.92;1.72)	0.17, (-0.56;0.91)	1.13, (0.83;1.55)	0.37, (-1.32;2.05)	0.28, (-0.81;1.36)	0.09, (-0.46;0.64)	0.08, (-0.21;0.37)
	At 6, 11 and 18y	1.69, (1.09;2.62)	0.28, (-0.70;1.25)	1.13, (0.75;1.71)	0.53, (-1.71;2.77)	-0.12, (-1.56;1.31)	0.17, (-0.56;0.90)	0.11, (-0.28;0.50)
	*Females*	*P* =0.014	*P* =0.467	*P* =0.081	*P* =0.450	*P* =0.329	*P* =0.373	*P* =0.437
	Never	Ref.	Ref.	Ref.	Ref.	Ref.	Ref.	Ref.
	At 6 and/or 11y	1.47, (0.97;2.24)	0.84, (-0.32;2.00)	1.34, (0.90;2.00)	1.43, (-0.95;3.81)	0.36, (-0.84;1.56)	0.64, (-0.25;1.53)	0.20, (-0.18;0.58)
	At 18y only	1.19, (0.93;1.53)	0.31, (-0.40;1.01)	1.27, (0.99;1.63)	0.52, (-0.93;1.97)	0.64, (-0.09;1.37)	0.15, (-0.39;0.70)	0.15, (-0.08;0.38)
	At 6, 11 and 18y	1.78, (1.20;2.62)	0.49, (-0.56;1.53)	1.48, (1.03;2.12)	1.44, (-0.71;3.59)	-0.05, (-1.13;1.03)	0.53, (-0.27;1.34)	-0.05, (-0.39;0.30)
Internalizing disorders	*Males*	*P* =0.655	*P* =0.932	*P* =0.808	*P* =0.961	*P* =0.821	*P* =0.951	*P* =0.887
	Never	Ref.	Ref.	Ref.	Ref.	Ref.	Ref.	Ref.
	At 6 and/or 11y	1.22, (0.83;1.80)	0.26, (-0.64;1.16)	0.89, (0.60;1.32)	0.56, (-1.50;2.63)	-0.34, (-1.66;0.99)	0.19, (-0.48;0.87)	0.06, (-0.30;0.42)
	At 18y only	1.08, (0.78;1.50)	-0.03, (-0.81;0.74)	1.07, (0.77;1.48)	0.12, (-1.66;1.91)	-0.24, (-1.38;0.90)	0.04, (-0.54;0.62)	-0.08, (-0.39;0.23)
	At 6, 11 and 18y	0.83, (0.45;1.55)	-0.22, (-1.76;1.33)	1.23, (0.65;2.31)	-0.01, (-3.56;3.55)	-0.93, (-3.21;1.35)	-0.06, (-1.22;1.10)	-0.15, (-0.77;0.46)
	*Females*	*P* = 0.474	*P=* 0.883	*P =* 0.570	*P =* 0.608	*P =* 0.929	*P =* 0.620	*P =* 0.876
	Never	Ref.	Ref.	Ref.	Ref.	Ref.	Ref.	Ref.
	At 6 and/or 11y	1.26, (0.79;2.02)	0.23, (-1.09;1.54)	1.14, (0.72;1.79)	0.03, (-2.66;2.73)	-0.38, (-1.74;0.97)	0.19, (-0.82;1.20)	0.04, (-0.40;0.47)
	At 18y only	1.11, (0.87;1.42)	-0.01, (-0.70;0.69)	1.11, (0.87;1.42)	0.14, (-1.29;1.56)	0.08, (-0.63;0.80)	0.02, (-0.51;0.55)	-0.03, (-0.26;0.20)
	At 6, 11 and 18y	1.35, (0.84;2.15)	0.48, (-0.81;1.77)	1.32, (0.85;2.06)	1.81, (-0.83;4.45)	-0.10, (-1.43;1.23)	0.65, (-0.34;1.64)	-0.17, (-0.59;0.26)
Externalizing disorders	*Males*	*P =* 0.002	*P =* 0.404	*P =* 0.388	*P =* 0.406	*P =* 0.321	*P =* 0.399	*P =* 0.720
	Never	Ref.	Ref.	Ref.	Ref.	Ref.	Ref.	Ref.
	At 6 and/or 11y	1.69, (1.04;2.76)	0.63, (-0.45;1.70)	1.32, (0.86;2.04)	1.05, (-1.42;3.52)	-0.09, (-1.68;1.49)	0.42, (-0.38;1.23)	0.20, (-0.23;0.63)
	At 18y only	1.82, (1.22;2.71)	-0.30, (-1.16;0.56)	0.91, (0.63;1.33)	-0.84, (-2.82;1.14)	-0.03, (-1.30;1.24)	-0.29, (-0.94;0.36)	-0.01, (-0.35;0.34)
	At 6, 11 and 18y	1.87, (0.80;4.32)	-0.88, (-2.66;0.90)	0.66, (0.28;1.52)	-2.45, (-6.54;1.65)	-2.51, (-5.13;0.12)	-0.65, (-1.99;0.68)	-0.23, (-0.94;0.48)
	*Females*	*P =* 0.027	*P =* 0.025	*P =* 0.006	*P =* 0.057	*P =* 0.040	*P =* 0.053	*P =* 0.029
	Never	Ref.	Ref.	Ref.	Ref.	Ref.	Ref.	Ref.
	At 6 and/or 11y	1.72, (0.94;3.15)	1.86, (0.27;3.45)	1.87, (1.09;3.22)	3.57, (0.31;6.83)	1.21, (-0.43;2.85)	1.35, (0.13;2.57)	0.51, (-0.02;1.03)
	At 18y only	1.37, (1.00;1.90)	0.72, (-0.17;1.61)	1.25, (0.92;1.70)	1.30, (-0.53;3.13)	1.21, (0.29;2.13)	0.39, (-0.29;1.08)	0.32, (0.03;0.62)
	At 6, 11 and 18y	2.80, (0.79;9.87)	2.38, (-0.51;5.28)	3.69, (1.27;10.69)	3.97, (-1.96;9.91)	0.89, (-2.10;3.88)	1.74, (-0.48;3.96)	0.64, (-0.31;1.59)

UPF: ultra-processed food; g: grams; BMI: body mass index; kg/m^2^: kilograms per meter squared; FM: fat mass; kg: kilograms; FFM: fat free mass; FMI: fat mass index; FFMI: fat free mass index; β: beta regression coefficient; OR: odds ratio; 95%CI: 95% confidence interval.

a Highest tertile of daily UPF consumption.

The males who have had EXT disorders “at 6 and/or 11 years” and “at 18 years only” had higher odds of higher daily consumption of UPF at 18 years than those who have never had. Among the females, those who have had EXT disorders “at 6 and/or 11 years” had higher BMI and higher odds of excessive weight at 18 years, whereas those who had EXT disorders “only at 18 years” had higher odds of being at the highest tertile of daily UPF consumption, higher FFM and higher FFMI. The females with EXT disorders “at 6, 11, and 18 years” had higher odds of excessive weight at 18 years.

Table 6 presents the results of the adjusted analyses. The association between ANY disorder and the highest tertile of daily UPF consumption observed among the females in unadjusted analysis was lost after allowing for confounders. The odds of excessive weight was higher for females with ANY disorders at any age (OR: 1.89; 95%CI 1.13–3.17). The females with ANY disorder “only at 18 years” presented higher FFM (β coefficient: 0.81; 95%CI 0.29–1.33) than those who had never had ANY disorders. INT disorders at any age remained not associated with any of the outcomes in both sexes.

**Table 6:** Adjusted analysis of the association between daily UPF consumption (highest tertile vs. intermediate and lowest tertiles), BMI, excessive weight, and body composition at 18 years with any, internalizing and externalizing disorders. (N=2,722)

Disorders		Food consumption and body composition parameters						
		Highest tertile of daily UPF consumption^a^	BMI (kg/m^2^)	Excessive weight	FM (kg)	FFM (kg)	FMI (kg/m^2^)	FFMI (kg/m^2^)
		OR (95%CI)	β (95%CI)	OR (95%CI)	β (95%CI)	β (95%CI)	β (95%CI)	β (95%CI)
Any disorder	*Males*	*p* = 0.470^1^	*p* = 0.380^3^	*p* = 0.358^6^	*p* = 0.173^9^	*p* = 0.787^12^	*p* = 0.135^14^	*p* = 0.310^16^
	Never	Ref.	Ref.	Ref.	Ref.	Ref.	Ref.	Ref.
	At 6 and/or 11y	0.96, (0.68;1.37)	0.37, (-0.29;1.02)	1.24, (0.84;1.84)	2.00, (0.21;3.78)	-0.11, (-0.94;0.72)	0.68, (0.10;1.26)	0.15, (-0.12;0.41)
	At 18y only	1.22, (0.86;1.72)	-0.28, (-0.88;0.32)	1.00, (0.69;1.45)	0.09, (-1.55;1.74)	-0.40, (-1.17;0.37)	-0.02, (-0.56;0.52)	-0.14, (-0.38;0.10)
	At 6, 11 and 18y	1.32, (0.82;2.12)	0.26, (-0.55;1.07)	1.47, (0.90;2.41)	0.32, (-1.90;2.53)	-0.06, (-1.11;0.99)	0.10, (-0.63;0.82)	0.10, (-0.24;0.44)
	*Females*	*p* = 0.109^2^	*p* = 0.653^4^	*p* = 0.045^7^	*p* = 0.405^10^	*p* = 0.023^13^	*p* = 0.532^15^	*p* = 0.328^17^
	Never	Ref.	Ref.	Ref.	Ref.	Ref.	Ref.	Ref.
	At 6 and/or 11y	1.11, (0.68;1.82)	0.44, (-0.64;1.52)	1.76, (0.99;3.13)	0.65, (-1.68;2.98)	0.48, (-0.38;1.34)	0.17, (-0.72;1.06)	0.15, (-0.14;0.44)
	At 18y only	1.27, (0.95;1.68)	-0.24, (-0.88;0.40)	1.16, (0.82;1.65)	-0.51, (-1.88;0.86)	0.81, (0.29;1.33)	-0.29, (-0.81;0.23)	0.15, (-0.02;0.32)
	At 6, 11 and 18y	1.65, (1.06;2.58)	0.01, (-1.00;1.02)	1.89, (1.13;3.17)	1.26, (-0.91;3.43)	0.45, (-0.33;1.22)	0.20, (-0.64;1.03)	0.01, (-0.25;0.27)
Internalizing disorders	*Males*	*p* = 0.735^1^	*p* = 0.503^3^	*p* = 0.592^8^	*p* = 0.791^9^	*p* = 0.330^12^	*p* = 0.814^14^	*p* = 0.358^16^
	Never	Ref.	Ref.	Ref.	Ref.	Ref.	Ref.	Ref.
	At 6 and/or 11y	0.92, (0.61;1.38)	-0.42, (-1.18;0.34)	0.83, (0.52;1.33)	-0.85, (-2.89;1.18)	-0.49, (-1.45;0.47)	-0.26, (-0.92;0.41)	0.09, (-0.22;0.40)
	At 18y only	1.06, (0.74;1.52)	-0.36, (-1.00;0.28)	0.94, (0.64;1.39)	0.32, (-1.45;2.08)	-0.69, (-1.51;0.13)	0.10, (-0.47;0.68)	-0.21, (-0.47;0.05)
	At 6, 11 and 18y	0.71, (0.36;1.40)	0.23, (-1.06;1.52)	1.51, (0.70;3.29)	-0.71, (-4.31;2.89)	-0.44, (-2.10;1.23)	-0.25, (-1.43;0.93)	-0.05, (-0.57;0.47)
	*Females*	*p* = 0.253^2^	*p* = 0.739^4^	*p* = 0.563^7^	*p* = 0.829^10^	*p* = 0.275^13^	*p* = 0.871^15^	*p* = 0.507^17^
	Never	Ref.	Ref.	Ref.	Ref.	Ref.	Ref.	Ref.
	At 6 and/or 11y	1.26, (0.73;2.16)	0.16, (-1.05;1.38)	1.40, (0.74;2.63)	-0.40, (-3.06;2.26)	0.29, (-0.67;1.26)	-0.07, (-1.07;0.92)	0.18, (-0.15;0.50)
	At 18y only	1.32, (0.99;1.74)	-0.30, (-0.93;0.33)	1.09, (0.78;1.52)	-0.43, (-1.79;0.93)	0.50, (-0.01;1.01)	-0.22, (-0.73;0.30)	0.07, (-0.10;0.24)
	At 6, 11 and 18y	1.27, (0.75;2.14)	-0.34, (-1.61;0.92)	1.44, (0.75;2.75)	0.74, (-1.95;3.43)	0.34, (-0.61;1.29)	0.01, (-1.03;1.05)	-0.11, (-0.43;0.21)
Externalizing disorders	*Males*	*p* = 0.144^1^	*p* = 0.079^5^	*p* = 0.232^8^	*p* = 0.003^9^	*p* = 0.897^12^	*p* = 0.002^14^	*p* = 0.654^16^
	Never	Ref.	Ref.	Ref.	Ref.	Ref.	Ref.	Ref.
	At 6 and/or 11y	1.24, (0.73;2.11)	1.24, (0.11;2.36)	1.74, (1.02;2.96)	4.45, (1.85;7.06)	-0.01, (-1.16;1.15)	1.47, (0.63;2.31)	-0.02, (-0.37;0.32)
	At 18y only	1.65, (1.07;2.56)	-0.51, (-1.32;0.30)	1.03, (0.67;1.60)	-1.17, (-3.05;0.71)	0.10, (-0.81;1.01)	-0.40, (-1.02;0.21)	-0.02, (-0.30;0.25)
	At 6, 11 and 18y	1.15, (0.47;2.79)	0.10, (-1.89;2.09)	1.26, (0.49;3.30)	-0.64, (-4.94;3.66)	-0.73, (-2.71;1.24)	-0.19, (-1.60;1.21)	-0.38, (-0.97;0.21)
	*Females*	*p* = 0.534^2^	*p* = 0.047^4^	*p* = 0.001^7^	*p* = 0.004^11^	*p* = 0.033^13^	*p* = 0.038^15^	*p* = 0.051^17^
	Never	Ref.	Ref.	Ref.	Ref.	Ref.	Ref.	Ref.
	At 6 and/or 11y	1.21, (0.61;2.39)	1.70, (0.18;3.23)	3.39, (1.56;7.36)	4.74, (1.42;8.06)	0.24, (-0.89;1.38)	1.53, (0.28;2.79)	0.09, (-0.29;0.47)
	At 18y only	1.22, (0.85;1.76)	0.46, (-0.34;1.26)	1.20, (0.77;1.81)	1.35, (-0.36;3.05)	0.89, (0.25;1.54)	0.24, (-0.41;0.90)	0.29, (0.08;0.51)
	At 6, 11 and 18y	1.99, (0.49;8.06)	2.50, (-0.55;5.56)	7.08, (1.69;29.59)	6.38, (0.14;12.63)	1.42, (-0.75;3.59)	2.11, (-0.40;4.63)	0.35, (-0.33;1.04)

UPF: ultra-processed food; g: grams; BMI: body mass index; kg/m^2^: kilograms per meter squared; FM: fat mass; kg: kilograms; FFM: fat free mass; FMI: fat mass index; FFMI: fat free mass index; β: beta regression coefficient; OR: odds ratio; 95%CI: 95% confidence interval.

a Highest tertile of daily UPF consumption.

1 Adjusted for family income at birth, maternal education and age, adolescent skin color, and intelligence quotient (IQ) at 6 years.

2 Adjusted for maternal education, age and parity, intrauterine growth, breastfeeding pattern at 3 months, adolescent skin color, and IQ at 6 years.

3 Adjusted for maternal education and parity, intrauterine growth, and duration of breastfeeding.

4 Adjusted for maternal education, intrauterine growth, adolescent skin color, screen time, and physical activity at 6 years.

5 Adjusted for maternal education and parity, intrauterine growth, duration of breastfeeding and physical activity at 6 years.

6 Adjusted for maternal age and parity, intrauterine growth, adolescent skin color, and duration of breastfeeding.

7 Adjusted for intrauterine growth, adolescent skin color, and physical activity at 6 years.

8 Adjusted for maternal age and parity, intrauterine growth, and duration of breastfeeding.

9 Adjusted for maternal education, age and parity, adolescent skin color, duration of breastfeeding, and physical activity at 6 years.

10 Adjusted for intrauterine growth, screen time, and physical activity at 6 years.

11 Adjusted for intrauterine growth, duration of breastfeeding, screen time, and physical activity at 6 years.

12 Adjusted for maternal education and age, intrauterine growth, and duration of breastfeeding.

13 Adjusted for maternal education and age, intrauterine growth, and adolescent skin color.

14 Adjusted for maternal age and parity, adolescent skin color, duration of breastfeeding, and physical activity at 6 years.

15 Adjusted for maternal education, intrauterine growth, screen time, and physical activity at 6 years.

16 Adjusted for maternal education and age, intrauterine growth, duration of breastfeeding, adolescent skin color, and screen time at 6 years.

17 Adjusted for intrauterine growth and adolescent skin color. The adjusted model included the measure of the outcome variable collected at the 6-year follow-up (period of onset of the exposure of interest).

FM and FMI were higher in males with EXT disorders “at 6 and/or 11 years” than among those who never had EXT disorders. Among the females, those with EXT “at 6 and/or 11 years” and “at 6, 11 and 18 years” presented increased odds of excessive weight in comparison to those who never had EXT disorders. Those with EXT disorders “at 6, 11 and 18 years”, for instance, had an odds seven times higher of excessive weight at 18 years (OR: 7.08; 95%CI 1.69–20.59) than those who have never had EXT disorders. Higher BMI (β coefficient: 1.70; 95%CI 0.18–3.23), FM (β coefficient: 4.74; 95%CI 1.42–8.06), and FMI (β coefficient: 1.53; 95%CI 0.528–2.79) were seen among the females with EXT disorders “at 6 and/or 11 years”. FM was also greater in females with EXT disorders “at 6, 11 and 18 years”, whereas FFM was higher in those with EXT disorders “only at 18,” in comparison to their counterparts who have never had EXT disorders.

The results of the analyses to check for reverse causality are displayed on Table S1. Among males there was no association between any of the exposures and mental health. Among the females the odds ratio for any disorder was higher among those with excessive weight “at 18 years only”, whereas the highest tertile of FMI “at 6 and/or 11 years” was protective. The highest tertile of FM “at 18 years only” was protective against INT disorders.

Tables S2 and S3 show respectively the unadjusted and adjusted results of the analyses after the exclusion of children with excessive weight at 6 or 11 years. The results from the unadjusted analyses for the association of ANY and INT disorders with the outcomes were similar to the results shown in Table 5. For EXT disorders, there was no association with daily UPF consumption for males and females, the association with BMI, excessive weight and FFMI was similar to that shown in Table 5, and new associations emerged with BMI, FM and FMI among the males and with FFM and FMI among the females. The results from the adjusted analyses were similar to those obtained from the analyses shown in Table 6, except for ANY disorders that were not associated with any of the outcomes.

## Discussion

This study showed that the association of having mental disorders in childhood and adolescence with the outcomes at 18 years varies with sex, with greater repercussion among the females. Our findings provide evidence that EXT disorders at childhood and/or early adolescence may increase the risk for future greater adiposity among adolescents from both sexes. The observed associations extend along a *continuum,* ranging from higher BMI, increased odds of excessive weight and greater FM and FMI. Previous studies showed that higher scores on ADHD symptoms at 11 years predicted a higher BMI at 15 years, and body fat composition in adulthood [37], and that depression and anxiety are underlying mechanisms in the association between ever having ADHD and obesity in adolescents [38].

The differential association of mental disorders and adiposity according to sex was also detected in a study with twins aged 16–17 years to examine sex-specific phenotypic correlations between the presence of ADHD symptoms and overweight/obese status [39]. Although present in both sexes, the positive association between ADHD symptoms and overweight/obese status was stronger in females than males. ADHD symptoms and overweight/obese status were highly heritable and shared genetic effects explained most of the phenotypic correlations in females.

Only a few studies explored the temporal sequence between mental disorders in normal-weight children or controlled for nutritional status at the beginning of the follow-up period. One of these studies was carried out in a random sample of The Health and Lifestyle Survey, which is a representative sample of the United Kingdom population aged 18 years and over [40]. The participants had an assessment of adiposity and mental health both at baseline and re-survey seven years later. Subjects with baseline obesity were excluded from analyses of the influence of baseline common mental disorder on the onset of obesity at re-survey; and in analyses of the influence of adiposity on the onset of common mental disorder, subjects with common mental disorder at baseline were excluded from the analyses. Participants with common mental disorder at baseline experienced greater odds of subsequently becoming overweight (women, OR: 1.30, 95%CI 1.03–1.64; men, OR: 1.05, 95%CI 0.81–1.38) and obese (women, OR: 1.26, 95%CI 0.82–1.94; men, OR: 2.10, 95%CI 1.23–3.55) than those who were free of common mental disorder. Similarly, having baseline common mental health disorder was related to a greater risk of developing moderate (OR: 1.57, 95%CI 1.21–2.04) and severe (OR: 1.48, 95%CI 1.09–2.01) abdominal obesity (women only). Inversely, baseline general or abdominal obesity was not associated with the risk of future common mental disorder, thus suggesting that the direction of association between common mental disorders and adiposity is from common mental disorder to increased future risk of adiposity as opposed to the inverse.

The British Whitehall II study, whose target population was all London-based office staff, aged 35–55 years, at study baseline in 1985–1988, followed-up the participants with a medical examination that have taken place on three occasions over a 19-year period [41]. In models adjusted for age, sex, and BMI at baseline, OR for obesity at the most recent screening were 1.33 (95%CI 1.00–1.77), 1.64 (95%CI 1.13–2.36), and 2.01 (95%CI 1.21–3.34) for participants with common mental disorder at one, two, or three preceding screenings, compared with people free from common mental disorder (*p* for trend < 0.001). These associations remained after adjustment for baseline characteristics related to mental health and exclusion of participants who were obese at baseline. In addition, obesity predicted future risk of common mental disorder, but the association was lost when people with common mental disorder at baseline were excluded.

The vast majority of the literature on the association of child and adolescent's mental health and behavior with excessive weight is based in studies investigating the co-occurrence of both problems. Most of the studies investigate depressive symptoms as a consequence of excessive weight and report a positive association between overweight, obesity and depression and/or anxiety. Despite this, however, we did not find any association between INT disorders and BMI, excessive weight, or body composition in both sexes. In the same way, despite the tendency for externalizing and internalizing problems to be comorbid with one another, strongly suggesting a shared vulnerability across domains [42,43], except for higher FFM in females exposed to ANY disorders “only at 18” (thus in a cross-sectional fashion), we find no association between ANY disorders and any of the adiposity outcomes.

Normative stress, defined as frequent stressors that occur in almost all adolescents in some aspect, regardless of gender, race, ethnicity, region, or socioeconomic status, and which include pubertal changes (changes in hormones, physical growth), cognitive and psychological changes (need for more social autonomy, search for identity), and adjustments in school and social contexts (academic pressures, peer problems, family conflict) was found to contribute to the development of EXT problems in adolescents [44]. Besides normative stress, general family stress, stressful family events (such as moving), and parent-child conflict predict adolescent EXT behaviors [45,46]. The relationship between stress and health is particularly evident in adult populations. Increased experience of stress has been associated with an increase in the consumption of high-calorie foods [47]. Stress has also been linked to an increase in consumption of between-meal snacks and reduced consumption of low-calorie high-nutrient foods like fruit and vegetables [48]. As demonstrated in other studies, these stress related eating behaviors can have deleterious effects on health by increasing body adiposity [49], particularly in abdominal areas [50] and subsequently heighten the risk of becoming overweight or obese [51]. A systematic review and meta-analysis aiming to identify whether stress is associated with healthy and unhealthy eating behaviors in children and adolescents aged 8–18 years found that the effect of stress on unhealthy eating may begin as early as 8 or 9 years old [52].

### Strengths and limitations

The present study has several strengths: a large population-based sample with information on mental health gathered from middle childhood to late adolescence through the use of internationally recognized instruments, administered by highly trained psychologists that ensured good quality data. Due to the longitudinal design and various follow-ups, information about exposure, mediators, outcomes, and even confounders are unlikely to present recall bias. For UPF consumption, however, the recall period for the self-administrated FFQ was 12 months. A previous study assessed the validity of the FFQ developed for the Pelotas Birth Cohort Studies against two 24-hour recalls taken as the gold standard, administered 14–28 days apart [53]. Although the relative validity was weak for specific nutrients and food groups, the FFQ showed to provide a reasonable dietary intake assessment for habitual food consumption. Nonetheless, FFQ relies on memory and is subject to under and overreporting.

Most studies assessing the association between mental disorders and nutritional status in childhood and adolescence have been conducted in high-income countries (HIC) [9]. Thus, we could examine whether the findings obtained in HIC apply to a middle-income country like Brazil. Also, we examined the association of mental disorders alongside the *continuum* of the putative mechanism through which mental disorders may increase the prevalence of overweight and obesity. Starting with the analysis of UPF consumption, subsequently the study explored the association with BMI and excessive weigh and finally with body composition parameters. Reverse causality was checked and there was no consistency between findings from the analyses when mental health was the exposure and when mental health was the outcome of interest, thus suggesting that this type of bias would not be affecting our results. The similarity of the results after allowing for the outcome variable at the baseline of the exposure period (6 years) or after the exclusion of children with excessive weight at 6 or 11 years reinforces the value of both measures to assess the association of early exposures on outcomes later in life.

Nonetheless, some limitations must be kept in mind when interpreting our findings. First, some diagnoses (like ADHD) can only be made when there is evidence that the disorder is present in two or more settings (usually home and school), and other problems may be highly situational, for example, severe conduct problems may be present at school but not at home, or vice versa. At 6 and 11 years we collected information only from the mother. Second, different informants were used to measure mental disorders in childhood, early adolescence and at 18 years, meaning that it was difficult to assess whether there was any information bias. Mothers may give a convincing account of their child being depressed, whereas adolescents may better describe worries or antisocial activities that they have successfully hidden from the adults around them. Then, parents may under- or over-report child mental health and behaviors, thus diminishing or increasing the strength of the associations. Also, although we adjusted the analysis for several measured confounders, as this was an observational study, the presence of unmeasured or residual confounders cannot unequivocally be ruled out. The results of our study may hold true in other urban settings with similar demographic and socio-economic structure as the city of Pelotas.

## Conclusions

Our findings of a longitudinal association between EXT disorders and adiposity may have important implications for practice. Health care providers should be aware that within children and adolescents showing symptoms of EXT disorders, weight should be monitored carefully, aiming the prevention, early detection and co treatment of those at risk, thus ultimately contributing to reduce excessive weight prevalence in adolescence.

## Authorship

ISS conceived the study. ISS and IOB analyzed data. All authors were involved in writing the paper and had final approval of the submitted version.

## Ethical Standards Disclosure

This study was conducted according to the guidelines laid down in the Declaration of Helsinki, and all procedures involving study participants were approved by the Ethics Committee of the Faculty of Medicine of the Federal University of Pelotas (40602124, 889.753 and 5.210.484, respectively, for the 6, 11 and 18 years). Written informed consent was obtained from the mothers or legal guardians. At 11 and 18 years old, the participants also signed a free and informed consent form.

## CRediT authorship contribution statement

**Iná S. Santos:** Writing – review & editing, Writing – original draft, Supervision, Formal analysis, Conceptualization. **Isabel O. Bierhals:** Writing – review & editing, Writing – original draft, Formal analysis. **Luciana Tovo-Rodrigues:** Writing – review & editing, Writing – original draft, Supervision. **Aluísio JD Barros:** Writing – review & editing, Writing – original draft, Supervision. **Tiago Munhoz:** Writing – review & editing, Writing – original draft. **Marina Xavier Carpena:** Writing – review & editing, Writing – original draft. **Alicia Matijasevich:** Writing – review & editing, Writing – original draft, Supervision.

## Declaration of competing interest

The authors declare that they have no known competing financial interests or personal relationships that could have appeared to influence the work reported in this paper.

## Appendix Supplementary materials

Image, application 1

## Footnotes

Supplementary material associated with this article can be found in the online version at doi:10.1016/j.nut.2024.112527.
