# Supplementary material for: Mental health from childhood to adolescence predicts excessive weight and body composition at 18 years
Source: Nutrition. 2024 Oct;126:None. doi: 10.1016/j.nut.2024.112527 (PMC11413523; doi:10.1016/j.nut.2024.112527)
Supplement: Supplementary file 1 [file mmc1.docx]

**SUPPLEMENTARY MATERIAL**

| **Table S1.** Adjusted analysis for the association between any, internalizing and externalizing disorders at 18 years with higher UPF consumption, BMI and body composition parameters and excessive weight from childhood to adolescence. (N=2,722) | | | | | | |
| --- | --- | --- | --- | --- | --- | --- |
| **Scores** | ***Male*** | | | ***Female*** | | |
|  | **Any disorder** | **Internalizing disorder** | **Externalizing disorder** | **Any disorder** | **Internalizing disorder** | **Externalizing disorder** |
|  | **OR (95%CI)** | **OR (95%CI)** | **OR (95%CI)** | **OR (95%CI)** | **OR (95%CI)** | **OR (95%CI)** |
| **UPF (highest tertile^a^)** | p=0.243^1^ | p=0.671^2^ | p=0.102^3^ | p=0.110^4^ | p=0.072^5^ | p=0.302^6^ |
| Never | 1.00 | 1.00 | 1.00 | 1.00 | 1.00 | 1.00 |
| At 6 and/or 11y | 1.16 (0.86;1.55) | 1.07 (0.75;1.51) | 1.32 (0.91;1.92) | 1.17 (0.88;1.56) | 1.30 (0.97;1.75) | 1.08 (0.75;1.58) |
| At 18y only | 0.92 (0.62;1.38) | 1.26 (0.80;1.96) | 0.74 (0.42;1.31) | 0.76 (0.52;1.13) | 0.82 (0.54;1.23) | 0.69 (0.39;1.21) |
| At 6, 11 and 18y | 0.78 (0.53;1.14) | 0.92 (0.59;1.44) | 0.78 (0.46;1.33) | 0.87 (0.63;1.19) | 0.94 (0.68;1.31) | 0.81 (0.53;1.24) |
|  |  |  |  |  |  |  |
| **BMI (highest tertile^a^)** | p=0.563^1^ | p=0.932^1^ | p=0.160^7^ | p=0.828^8^ | p=0.659^9^ | p=0.484^6^ |
| Never | 1.00 | 1.00 | 1.00 | 1.00 | 1.00 | 1.00 |
| At 6 and/or 11y | 0.91 (0.60;1.39) | 0.91 (0.56;1.50) | 1.03 (0.60;1.77) | 0.92 (0.68;1.25) | 0.97 (0.71;1.32) | 1.17 (0.79;1.72) |
| At 18y only | 0.80 (0.52;1.23) | 0.95 (0.58;1.54) | 0.63 (0.39;1.18) | 0.86 (0.49;1.51) | 1.17 (0.66;2.08) | 0.55 (0.21;1.41) |
| At 6, 11 and 18y | 1.09 (0.82;1.45) | 1.07 (0.77;1.48) | 1.28 (0.89;1.84) | 1.06 (0.80;1.41) | 1.17 (0.88;1.56) | 1.01 (0.69;1.48) |
|  |  |  |  |  |  |  |
| **Excessive weight (yes)** | p=0.588^10^ | p=0.639^11^ | p=0.565^7^ | **p=0.017^8^** | p=0.056^12^ | p=0.215^13^ |
| Never | 1.00 | 1.00 | 1.00 | 1.00 | 1.00 | 1.00 |
| At 6 and/or 11y | 0.99 (0.73;1.35) | 0.99 (0.69;1.42) | 0.76 (0.50;1.15) | 0.78 (0.56;1.09) | 0.77 (0.55;1.09) | 0.71 (0.43;1.16) |
| At 18y only | 0.71 (0.31;1.62) | 1.22 (0.51;2.89) | 0.87 (0.32;2.34) | 1.57 (1.00;2.48) | 1.52 (0.96;2.39) | 1.43 (0.82;2.48) |
| At 6, 11 and 18y | 1.16 (0.86;1.56) | 1.22 (0.87;1.72) | 0.82 (0.55;1.22) | 1.23 (0.95;1.59) | 1.13 (0.87;1.47) | 1.06 (0.75;1.50) |
|  |  |  |  |  |  |  |
| **FM (highest tertile^a^)** | p=0.963^14^ | p=0.993^1^ | p=0.544^7^ | p=0.106^8^ | **p=0.020^12^** | p=0.171^6^ |
| Never | 1.00 | 1.00 | 1.00 | 1.00 | 1.00 | 1.00 |
| At 6 and/or 11y | 1.08 (0.67;1.73) | 0.98 (0.59;1.65) | 1.21 (0.68;2.15) | 0.79 (0.61;1.03) | 0.85 (0.64;1.12) | 1.05 (0;74;1.48) |
| At 18y only | 0.94 (0.64;1.39) | 0.97 (0.64;1.47) | 0.92 (0.56;1.52) | 0.96 (0.43;2.15) | 0.35 (0.13;0.96) | 2.60 (1.09;6.19) |
| At 6, 11 and 18y | 0.97 (0.72;1.31) | 0.95 (0.69;1.32) | 1.24 (0.85;1.80) | 1.28 (0.89;1.83) | 1.38 (0.96;1.99) | 0.91 (0.55;1.49) |
|  |  |  |  |  |  |  |
| **FFM (highest tertile^a^)** | p=0.845^1^ | p=0.619^1^ | p=0.111^7^ | p=0.152^8^ | p=0.606^12^ | p=0.193^6^ |
| Never | 1.00 | 1.00 | 1.00 | 1.00 | 1.00 | 1.00 |
| At 6 and/or 11y | 0.93 (0.72;1.21) | 0.86 (0.64;1.17) | 1.42 (1.02;1.96) | 1.26 (0.72;2.22) | 1.43 (0.81;2.52) | 1.09 (0.55;2.17) |
| At 18y only | - | - | - | 0.81 (0.60;1.10) | 0.96 (0.70;1.31) | 0.70 (0.46;1.06) |
| At 6, 11 and 18y | 1.08 (0.49;2.38) | 0.84 (0.32;2.22) | 1.23 (0.42;3.63) | 0.79 (0.61;1.03) | 1.00 (0.76;1.31) | 0.64 (0.52;1.05) |
|  |  |  |  |  |  |  |
| **FMI (highest tertile^a^)** | p=0.576^14^ | p=0.604^11^ | p=0.369^7^ | **p=0.030^15^** | p=0.054^12^ | p=0.873^13^ |
| Never | 1.00 | 1.00 | 1.00 | 1.00 | 1.00 | 1.00 |
| At 6 and/or 11y | 0.78 (0.47;1.28) | 0.74 (0.43;1.28) | 1.11 (0.62;1.98) | 0.76 (0.58;0.98) | 0.82 (0.63;1.07) | 1.01 (0.72;1.43) |
| At 18y only | 0.80 (0.54;1.17) | 0.81 (0.53;1.22) | 0.78 (0.47;1.28) | 0.60 (0.19;1.86) | 0.26 (0.06;1.19) | 1.69 (0.46;6.25) |
| At 6, 11 and 18y | 0.95 (0.70;1.29) | 0.86 (0.62;1.21) | 1.19 (0.81;1.73) | 1.37 (0.92;2.06) | 1.38 (0.92;2.08) | 0.93 (0.53;1.63) |
|  |  |  |  |  |  |  |
| **FFMI (highest tertile^a^)** | p=0.689^10^ | p=0.966^11^ | p=0.398^7^ | p=0.358^8^ | p=0.799^12^ | p=0.394^16^ |
| Never | 1.00 | 1.00 | 1.00 | 1.00 | 1.00 | 1.00 |
| At 6 and/or 11y | 0.88 (0.67;1.17) | 1.00 (0.73;1.37) | 1.22 (0.86;1.74) | 0.73 (0.46;1.17) | 0.94 (0.58;1.52) | 0.78 (0.41;1.47) |
| At 18y only | 0.42 (0.05;3.46) | 0.74 (0.09;6.26) | - | 0.92 (0.66;1.29) | 1.08 (0.77;1.52) | 0.69 (0.43;1.11) |
| At 6, 11 and 18y | 1.04 (0.67;1.62) | 0.89 (0.52;1.52) | 1.33 (0.76;2.30) | 0.82 (0.63;1.06) | 0.92 (0.71;1.20) | 0.81 (0.58;1.14) |
| UPF: ultra-processed food; BMI: body mass index; FM: fat mass; FFM: fat free mass; FMI: fat mass index; FFMI: fat free mass index; OR: odds ratio; 95%CI: 95% confidence interval.  ^a^ Highest tertile of the sample distribution at each age.  ^1^Adjusted for maternal age. ^2^Adjusted for maternal age, adolescent skin color, and intelligence quotient at 6 years. ^3^Adjusted for maternal age and parity, breastfeeding pattern at 3 months, and intelligence quotient at 6 years. ^4^Adjusted for maternal age and parity, adolescent skin color, and intelligence quotient at 6 years. ^5^Adjusted for maternal parity, breastfeeding pattern at 3 months, duration of breastfeeding, adolescent skin color, and intelligence quotient at 6 years. ^6^Adjusted for maternal parity. ^7^Adjusted for maternal age and parity, and breastfeeding pattern at 3 months. ^8^Adjusted for maternal parity and intelligence quotient at 6 years. ^9^Adjusted for maternal parity, breastfeeding pattern at 3 months, and intelligence quotient at 6 years. ^10^Adjusted for maternal age and parity. ^11^Adjusted for maternal age and adolescent skin color. ^12^Adjusted for maternal parity, breastfeeding pattern at 3 months, adolescent skin color, and intelligence quotient at 6 years. ^13^Adjusted for family income, maternal education and parity. ^14^Adjusted for maternal age and screen time at 6 years. ^15^ Adjusted for maternal age and parity, and intelligence quotient at 6 years. ^16^ Adjusted for maternal education and parity.  The adjusted model included the measure of the outcome variable collected at the 6-year follow-up (period of onset of the exposure of interest). | | | | | | |

| **Table S2.** Unadjusted analysis of the association between daily UPF consumption (highest tertile vs. intermediate and lowest tertiles), BMI, excessive weight, and body composition at 18 years with any, internalizing and externalizing disorders, excluding those individuals with excessive weight at 6 or 11 years of age. (N=1,845). | | | | | | | | |
| --- | --- | --- | --- | --- | --- | --- | --- | --- |
| **Disorders** | | **UPF consumption and body composition parameters** | | | | | | |
|  |  | **Highest tertile of consumption^a^** | **BMI (kg/m^2^)** | **Excessive weight** | **FM (kg)** | **FFM (kg)** | **FMI (kg/m^2^)** | **FFMI (kg/m^2^)** |
|  |  | **OR (95%CI)** | **β (95%CI)** | **OR (95%CI)** | **β (95%CI)** | **β (95%CI)** | **β (95%CI)** | **β (95%CI)** |
| **Any disorder** | ***Males*** | p=0.191 | p=0.133 | p=0.110 | p=0.280 | p=0.736 | p=0.166 | p=0.607 |
|  | Never | Ref. | Ref. | Ref. | Ref. | Ref. | Ref. | Ref. |
|  | At 6 and/or 11y | 1.09  (0.73;1.64) | 0.74  (0.11;1.38) | 1.83  (1.12;2.98) | 1.37  (-0.10;2.83) | -0.28  (-1.47;0.91) | 0.53  (0.05;1.01) | 0.22  (-0.10;0.54) |
|  | At 18y only | 1.15  (0.77;1.73) | 0.09  (-0.54;0.72) | 1.00  (0.57;1.75) | 0.14  (-1.30;1.58) | 0.24  (-0.93;1.42) | 0.04  (-0.44;0.51) | 0.05  (-0.26;0.37) |
|  | At 6, 11 and 18y | 1.85  (1.03;3.33) | -0.07  (-0.87;0.74) | 1.25  (0.64;2.45) | -0.31  (-2.15;1.54) | -0.66  (-2.16;0.83) | -0.07  (-0.67;0.54) | -0.01  (-0.41;0.40) |
|  | ***Females*** | **p=0.047** | p=0.249 | p=0.267 | p=0.248 | p=0.724 | p=0.113 | p=0.976 |
|  | Never | Ref. | Ref. | Ref. | Ref. | Ref. | Ref. | Ref. |
|  | At 6 and/or 11y | 1.39  (0.83;2.34) | 0.48  (-0.48;1.45) | 1.24  (0.70;2.21) | 0.78  (-1.18;2.75) | 0.11  (-1.05;1.27) | 0.41  (-0.35;1.18) | 0.07  (-0.30;0.43) |
|  | At 18y only | 1.24  (0.92;1.68) | -0.30  (-0.88;0.27) | 1.16  (0.82;1.66) | -0.66  (-1.83;0.51) | 0.40  (-0.29;1.09) | -0.32  (-0.78;0.13) | 0.02  (-0.20;0.24) |
|  | At 6, 11 and 18y | 1.89  (1.16;3.07) | 0.41  (-0.44;1.27) | 1.64  (1.01;2.67) | 0-.90  (-0.84;2.64) | 0.10  (-0.92;1.12) | 0.36  (-0.32;1.03) | 0.05  (-0.27;0.38) |
| **Internalizing disorders** | ***Males*** | p=0.996 | p=0.751 | p=0.464 | p=0.785 | p=0.380 | p=0.857 | p=0.828 |
|  | Never | Ref. | Ref. | Ref. | Ref. | Ref. | Ref. | Ref. |
|  | At 6 and/or 11y | 0.95  (0.59;1.52) | -0.40  (-1.15;0.35) | 0.66  (0.32;1.36) | -0.90  (-2.61;0.81) | -1.07  (-2.46;0.32) | -0.25  (-0.81;0.31) | -0.15  (-0.52;0.22) |
|  | At 18y only | 1.02  (0.66;1.57) | -0.14  (-0.81;0.53) | 1.02  (0.58;1.78) | -0.10  (-1.64;1.43) | -0.01  (-1.25;1.25) | -0.04  (-0.55;0.46) | -0.09  (-0.43;0.24) |
|  | At 6, 11 and 18y | 1.00  (0.44;2.27) | -0.21  (-1.50;1.07) | 1.63  (0.65;4.08) | -0.24  (-3.18;2.70) | -1.16  (-3.55;1.23) | -0.09  (-1.05;0.88) | -0.12  (-0.77;0.52) |
|  | ***Females*** | p=0.544 | p=0.075 | p=0.575 | p=0.188 | p=0.735 | p=0.112 | p=0.298 |
|  | Never | Ref. | Ref. | Ref. | Ref. | Ref. | Ref. | Ref. |
|  | At 6 and/or 11y | 1.19  (0.67;2.12) | 0.36  (-0.72;1.44) | 1.03  (0.54;1.98) | 0.54  (-1.66;2.74) | -0.08  (-1.37;1.21) | 0.33  (-0.53;1.18) | 0.04  (-0.37;0.44) |
|  | At 18y only | 1.18  (0.87;1.59) | -0.60  (-1.17;0.04) | 0.93  (0.66;1.32) | -0.98  (-2.13;0.17) | -0.31  (-0.99;0.36) | -0.41  (-0.86;0.03) | -0.19  (-0.40;0.02) |
|  | At 6, 11 and 18y | 1.40  (0.77;2.53) | 0.44  (-0.64;1.52) | 1.48  (0.81;2.70) | 0.95  (-1.24;3.15) | 0.31  (-0.98;1.60) | 0.39  (-0.47;1.24) | 0.05  (-0.36;0.46) |
| **Externalizing disorders** | ***Males*** | p=0.065 | **p=0.007** | p=0.058 | **p=0.008** | p=0.828 | **p=0.003** | p=0.391 |
|  | Never | Ref. | Ref. | Ref. | Ref. | Ref. | Ref. | Ref. |
|  | At 6 and/or 11y | 1.38  (0.78;2.47) | 1.40  (0.55;2.25) | 2.15  (1.19;3.86) | 2.89  (0.95;4.84) | 0.46  (-1.13;2.05) | 1.04  (0.41;1.68) | 0.36  (-0.07;0.79) |
|  | At 18y only | 1.53  (0.93;2.51) | -0.16  (-0.88;0.55) | 0.80  (0.41;1.55) | -0.34  (-1.97;1.29) | 0.39  (-0.94;1.73) | -0.14  (-0.68;0.39) | -0.02  (-0.38;0.34) |
|  | At 6, 11 and 18y | 2.93  (0.87;9.86) | -0.63  (-2.02;0.76) | 0.60  (0.14;2.58) | -2.54  (-5.72;0.64) | -0.63  (-3.23;1.97) | -0.81  (-1.85;0.23) | 0.18  (-0.52;0.87) |
|  | ***Females*** | p=0.060 | **p=0.010** | **p=0.006** | p=0.069 | p=0.285 | **p=0.031** | **p=0.045** |
|  | Never | Ref. | Ref. | Ref. | Ref. | Ref. | Ref. | Ref. |
|  | At 6 and/or 11y | 2.02  (0.91;4.50) | 1.95  (0.62;3.27) | 2.49  (1.25;4.99) | 3.28  (0.58;5.98) | 0.91  (-0.68;2.49) | 1.40  (0.36;2.45) | 0.54  (0.04;1.04) |
|  | At 18y only | 1.43  (0.95;2.14) | 0.01  (-0.73;0.74) | 1.25  (0.81;1.94) | 0.05  (-1.44;1.55) | 0.59  (-0.29;1.47) | -0.08  (-0.66;0.50) | 0.08  (-0.20;0.36) |
|  | At 6, 11 and 18y | 2.60  (0.56;12.12) | 2.19  (-0.17;4.55) | 4.70  (1.42;15.62) | 3.04  (-1.77;7.84) | 1.58  (-1.24;4.40) | 1.32  (-0.55;3.18) | 0.87  (-0.02;1.76) |
| UPF: ultra-processed food; g: grams; BMI: body mass index; kg/m^2^: kilograms per meter squared; FM: fat mass; kg: kilograms; FFM: fat free mass; FMI: fat mass index; FFMI: fat free mass index; β: beta regression coefficient; OR: odds ratio; 95%CI: 95% confidence interval.  ^a^ Highest tertile of daily UPF consumption. | | | | | | | | |

| **Table S3.** Adjusted analysis of the association between daily UPF consumption (highest tertile vs. intermediate and lowest tertiles), BMI, excessive weight, and body composition at 18 years with any, internalizing and externalizing disorders, excluding those individuals with excessive weight at 6 or 11 years of age. (N=1,845). | | | | | | | | |
| --- | --- | --- | --- | --- | --- | --- | --- | --- |
| **Disorders** | | **Food consumption and body composition parameters** | | | | | | |
|  |  | **Highest tertile of UPF consumption^a^** | **BMI (kg/m^2^)** | **Excessive weight** | **FM (kg)** | **FFM (kg)** | **FMI (kg/m^2^)** | **FFMI (kg/m^2^)** |
|  |  | **OR (95%CI)** | **β (95%CI)** | **OR (95%CI)** | **β (95%CI)** | **β (95%CI)** | **β (95%CI)** | **β (95%CI)** |
| **Any disorder** | ***Males*** | p=0.512^1^ | p=0.147^5^ | p=0.071^8^ | p=0.426^11^ | p=0.940^14^ | p=0.356^16^ | p=0.710^18^ |
|  | Never | Ref. | Ref. | Ref. | Ref. | Ref. | Ref. | Ref. |
|  | At 6 and/or 11y | 0.84  (0.54;1.30) | 0.76  (0.11;1.41) | 1.86  (1.13;3.07) | 1.44  (-0.26;3.15) | 0.21  (-0.72;1.15) | 0.51  (-0.05;1.07) | 0.12  (-0.16;0.40) |
|  | At 18y only | 1.04  (0.66;1.63) | 0.06  (-0.56;0.68) | 0.92  (0.51;1.67) | 0.38  (-1.25;2.00) | -0.15  (-1.04;0.75) | 0.13  (-0.41;0.66) | -0.06  (-0.33;0.21) |
|  | At 6, 11 and 18y | 1.46  (0.76;2.79) | 0.17  (-0.64;0.99) | 1.34  (0.68;2.65) | 0.38  (-1.72;2.47) | 0.09  (-1.08;1.27) | 0.10  (-0.60;0.79) | -0.08  (-0.44;0.27) |
|  | ***Females*** | p=0.596^2^ | p=0.334^6^ | p=0.139^9^ | p=0.141^12^ | p=0.141^15^ | p=0.193^17^ | p=0.813^19^ |
|  | Never | Ref. | Ref. | Ref. | Ref. | Ref. | Ref. | Ref. |
|  | At 6 and/or 11y | 0.94  (0.44;1.99) | 0.40  (-0.69;1.49) | 1.57  (0.79;3.11) | 0.44  (-1.80;2.67) | 0.43  (-0.49;1.36) | 0.21  (-0.68;1.11) | 0.08  (-0.24;0.41) |
|  | At 18y only | 1.20  (0.79;1.81) | -0.46  (-1.11;0.18) | 1.14  (0.74;1.75) | -1.16  (-2.50;0.17) | 0.61  (0.07;1.15) | -0.49  (-1.02;0.04) | 0.09  (-0.10;0.27) |
|  | At 6, 11 and 18y | 1.49  (0.76;2.95) | 0.08  (-0.95;1.10) | 1.90  (1.06;3.41) | 0.95  (-1.13;3.04) | 0.53  (-0.29;1.34) | 0.14  (-0.70;0.98) | 0.11  (-0.28;0.30) |
| **Internalizing disorders** | ***Males*** | p=0.644^1^ | p=0.816^5^ | p=0.436^10^ | p=0.838^11^ | p=0.982^14^ | p=0.837^16^ | p=0.768^14^ |
|  | Never | Ref. | Ref. | Ref. | Ref. | Ref. | Ref. | Ref. |
|  | At 6 and/or 11y | 0.73  (0.44;1.22) | -0.30  (-1.06;0.46) | 0.70  (0.34;1.45) | -0.67  (-2.59;1.25) | -0.06  (-1.16;1.03) | -0.23  (-0.87;0.41) | -0.09  (-0.42;0.24) |
|  | At 18y only | 0.99  (0.61;1.61) | -0.05  (-0.71;0.62) | 0.92  (0.51;1.65) | 0.40  (-1.35;2.16) | -0.20  (-1.15;0.76) | 0.13  (-0.46;0.71) | -0.14  (-0.43;0.14) |
|  | At 6, 11 and 18y | 0.77  (0.31;1.92) | 0.35  (-0.98;1.68) | 1.81  (0.71;4.63) | -0.50  (-3.91;2.90) | -0.08  (-2.00;1.84) | -0.15  (-1.28;0.97) | -0.03  (-0.62;0.56) |
|  | ***Females*** | p=0.289^3^ | p=0.133^6^ | p=0.883^9^ | p=0.282^12^ | p=0.433^15^ | p=0.301^17^ | p=0.629^19^ |
|  | Never | Ref. | Ref. | Ref. | Ref. | Ref. | Ref. | Ref. |
|  | At 6 and/or 11y | 1.10  (0.57;2.14) | 0.40  (-0.82;1.63) | 1.12  (0.50;2.47) | 0.19  (-2.38;2.77) | 0.24  (-0.78;1.26) | 0.19  (-0.81;1.20) | 0.13  (-0.22;0.49) |
|  | At 18y only | 1.41  (0.99;2.00) | -0.68  (-1.32;-0.05) | 0.99  (0.65;1.50) | -1.23  (-2.55;0.09) | -0.01  (-0.54;0.52) | -0.47  (-0.99;0.05) | -0.09  (-0.27;0.10) |
|  | At 6, 11 and 18y | 1.19  (0.61;2.31) | -0.34  (-1.64;0.96) | 1.40  (0.67;2.95) | 0.16  (-2.45;2.77) | 0.82  (-0.20;1.83) | -0.15  (-1.22;0.92) | -0.01  (-0.37;0.36) |
| **Externalizing disorders** | ***Males*** | p=0.524^4^ | p=0.073^7^ | **p=0.023^10^** | **p=0.009^11^** | p=0.943^14^ | **p=0.004^16^** | p=0.740^14^ |
|  | Never | Ref. | Ref. | Ref. | Ref. | Ref. | Ref. | Ref. |
|  | At 6 and/or 11y | 1.03  (0.54;1.95) | 1.27  (0.22;2.35) | 2.39  (1.31;4.39) | 3.78  (1.37;6.19) | 0.06  (-1.19;1.30) | 1.32  (0.54;2.11) | 0.08  (-0.28;0.45) |
|  | At 18y only | 1.28  (0.75;2.18) | -0.20  (-1.00;0.60) | 0.79  (0.39;1.59) | -0.48  (-2.28;1.32) | 0.20  (-0.83;1.23) | -0.14  (-0.74;0.46) | -0.08  (-0.39;0.23) |
|  | At 6, 11 and 18y | 2.24  (0.63;8.01) | -0.77  (-2.61;1.08) | 0.71  (0.16;3.11) | -2.06  (-5.83;1.72) | -0.49  (-2.57;1.58) | -0.76  (-2.01;0.48) | -0.28  (-0.92;0.36) |
|  | ***Females*** | p=0.684^3^ | **p=0.020^6^** | **p=0.003^9^** | **p=0.012^13^** | **p=0.030^15^** | **p=0.021^17^** | p=0.063^19^ |
|  | Never | Ref. | Ref. | Ref. | Ref. | Ref. | Ref. | Ref. |
|  | At 6 and/or 11y | 1.33  (0.54;3.30) | 2.28  (0.74;3.83) | 3.38  (1.47;7.81) | 4.71  (1.47;7.95) | 0.24  (-0.99;1.46) | 1.88  (0.61;3.15) | 0.20  (-0.23;0.63) |
|  | At 18y only | 1.26  (0.79;2.00) | 0.24  (-0.57;1.05) | 1.14  (0.67;1.94) | 0.21  (-1.47;1.89) | 0.72  (0.04;1.40) | -0.06  (-0.73;0.60) | 0.21  (-0.03;0.44) |
|  | At 6, 11 and 18y | 1.60  (0.27;9.57) | 1.99  (-1.06;5.05) | 6.29  (1.47;26.94) | 5.21  (-0.64;11.06) | 2.69  (0.33;5.05) | 1.53  (-0.98;4.03) | 0.83  (0.03;1.63) |
| UPF: ultra-processed food; g: grams; BMI: body mass index; kg/m^2^: kilograms per meter squared; FM: fat mass; kg: kilograms; FFM: fat free mass; FMI: fat mass index; FFMI: fat free mass index; β: beta regression coefficient; OR: odds ratio; 95%CI: 95% confidence interval.  ^a^ Highest tertile of daily UPF consumption.  ^1^Adjusted for maternal education and age, adolescent skin color, and intelligence quotient (IQ) at 6 years. ^2^Adjusted for maternal education and age, intrauterine growth, breastfeeding pattern at 3 months, adolescent skin color, physical activity, and IQ at 6 years. ^3^Adjusted for maternal education and age, intrauterine growth, breastfeeding pattern at 3 months, adolescent skin color, and IQ at 6 years. ^4^Adjusted for maternal education and age, breastfeeding pattern at 3 months, adolescent skin color, and IQ at 6 years. ^5^Adjusted for maternal education and parity, intrauterine growth and duration of breastfeeding. ^6^Adjusted for maternal education, adolescent skin color, intrauterine growth, screen time, and physical activity at 6y. ^7^Adjusted for maternal education and parity, intrauterine growth, duration of breastfeeding and physical activity at 6y. ^8^Adjusted for maternal age and parity, adolescent skin color, intrauterine growth, and duration of breastfeeding. ^9^Adjusted for adolescent skin color, intrauterine growth, and physical activity at 6y. ^10^Adjusted for maternal age and parity, intrauterine growth, and duration of breastfeeding. ^11^Adjusted for maternal education, age and parity, adolescent skin color, duration of breastfeeding, and physical activity at 6y. ^12^Adjusted for intrauterine growth, screen time, and physical activity at 6y. ^13^Adjusted for intrauterine growth, duration of breastfeeding, screen time, and physical activity at 6 years. ^14^Adjusted for maternal education and age, intrauterine growth, and duration of breastfeeding. ^15^Adjusted for maternal education and age, adolescent skin color, and intrauterine growth. ^16^Adjusted for maternal age and parity, adolescent skin color, duration of breastfeeding, and physical activity at 6y. ^17^Adjusted for maternal education, intrauterine growth, screen time, and physical activity at 6y. ^18^Adjusted for maternal education and age, intrauterine growth, duration of breastfeeding, and screen time at 6y. ^19^Adjusted for adolescent skin color, intrauterine growth, and IQ at 6y.  The adjusted model included the measure of the outcome variable collected at the 6-year follow-up (period of onset of the exposure of interest), with the exception of excessive weight, given that this analysis excluded individuals who were overweight at age 6 or 11. | | | | | | | | |
